# Supplementary material for: Integrin-based diffusion barrier separates membrane domains enabling the formation of microbiostatic frustrated phagosomes
Source: eLife. 2018 Mar 19;7:e34798. doi: 10.7554/eLife.34798 (PMC5897098; doi:10.7554/eLife.34798)
Supplement: Transparent reporting form [file elife-34798-transrepform.docx]

***eLife’s* transparent reporting form**

We encourage authors to provide detailed information *within their submission* to facilitate the interpretation and replication of experiments. If you have any questions, please contact us: [editorial@elifesciences.org](mailto:editorial@elifesciences.org).

**Sample-size estimation**

- You should state whether an appropriate sample size was computed when the study was being designed
- You should state the statistical method of sample size computation and any required assumptions
- If no explicit power analysis was used, you should describe how you decided what sample (replicate) size (number) to use

Please outline where this information can be found within the submission (e.g., page numbers or figure legends), or explain why this information doesn’t apply to your submission:

Power analysis was not used; the experiments were, for the most part, in vitro imaging determinations of individual host-pathogen interactions. The number of individual experiments and the number of determinations per experiment were selected to attain an estimate of the variance compatible with the statistical tests used, primarily student's t test.

**Replicates**

- You should report how often each experiment was performed
- You should include a definition of biological versus technical replication
- The data obtained should be provided and sufficient information should be provided to indicate the number of independent biological and/or technical replicates
- If you encountered any outliers, you should describe how these were handled
- Criteria for exclusion/inclusion of data should be clearly stated
- High-throughput sequence data should be uploaded before submission, with a private link for reviewers provided (these are available from both GEO and ArrayExpress)

Please outline where this information can be found within the submission (e.g., page numbers or figure legends), or explain why this information doesn’t apply to your submission:

Each type of experiment was performed a minimum of 3 separate times (biological replicates) and a minimum of 4-10 individual event determinations (equivalent, but not identical to technical replicates) were acquired for each of the experiments. Specific numbers for individual types of experiments are provided in the Results text (pages 5-20) and in the figure legends (pages 38-49). None of the data were considered outliers. No high-throughput sequencing was performed.

**Statistical reporting**

- Statistical analysis methods should be described and justified
- Raw data should be presented in figures whenever informative to do so (typically when N per group is less than 10)
- For each experiment, you should identify the statistical tests used, exact values of N, definitions of center, methods of multiple test correction, and dispersion and precision measures (e.g., mean, median, SD, SEM, confidence intervals; and, for the major substantive results, a measure of effect size (e.g., Pearson's r, Cohen's d)
- Report exact p-values wherever possible alongside the summary statistics and 95% confidence intervals. These should be reported for all key questions and not only when the p-value is less than 0.05.

Representative raw data are presented in all figures as individual images. Data were acquired from populations assumed to be normally distributed. The significance of any differences was established using student's t test for unpaired samples. p values are reported throughout the manuscript Results text (pages 5-10) and in the Figures (see Figures 2, 3, 4, 4-Supplement 1, 5, 9, 10 and 10-Supplement 2). Information detailing the statistical methods used is described in figure legends (pages 38-49).

Please outline where this information can be found within the submission (e.g., page numbers or figure legends), or explain why this information doesn’t apply to your submission:

(For large datasets, or papers with a very large number of statistical tests, you may upload a single table file with tests, Ns, etc., with reference to page numbers in the manuscript.)

**Additional data files (“source data”)**

- We encourage you to upload relevant additional data files, such as numerical data that are represented as a graph in a figure, or as a summary table
- Where provided, these should be in the most useful format, and they can be uploaded as “Source data” files linked to a main figure or table
- Include model definition files including the full list of parameters used
- Include code used for data analysis (e.g., R, MatLab)
- Avoid stating that data files are “available upon request”

Please indicate the figures or tables for which source data files have been provided:

Not applicable.
